# Correlation between estimated plasma volume status and extracellular volume ratio determined by bioelectrical impedance analysis in cardiovascular disease patients

**DOI:** 10.1016/j.ahjo.2026.100794

**Published:** 2026-05-11

**Authors:** Ryo Miyabe, Akinori Higaki, Keisho Kurokawa, Tomoaki Nishikawa, Rikako Horie, Yasuhisa Nakao, Tomoki Fujisawa, Yusuke Akazawa, Toru Miyoshi, Hiroshi Kawakami, Haruhiko Higashi, Shunsuke Tamaki, Kazuhisa Nishimura, Katsuji Inoue, Shuntaro Ikeda, Osamu Yamaguchi

**Affiliations:** Department of Cardiology, Pulmonology, Hypertension & Nephrology, Ehime University Graduate School of Medicine, Toon, Japan

## Abstract

This study investigated the correlation between estimated plasma volume status (ePVS), calculated from hematocrit, and the extracellular water–to–total body water (ECW/TBW) ratio measured by bioelectrical impedance analysis in 851 cardiovascular patients. We observed a significant positive correlation between ePVS and ECW/TBW (ρ = 0.491, *p* < 0.01). Notably, ECW/TBW demonstrated superior performance in identifying elevated B-type natriuretic peptide levels compared to ePVS (AUC: 0.84 [95% CI: 0.81–0.87] vs. 0.75 [95% CI: 0.71–0.80], *p* < 0.01), suggesting that BIA-derived indices may more accurately reflect cardiac congestion than formula-based plasma volume estimates.

## 1 Background

Estimated plasma volume status (ePVS) can be easily calculated from hemoglobin (Hb) and hematocrit (Hct) levels and is a well-validated prognostic indicator in patients with heart failure [1]. However, ePVS shows only a modest correlation with directly measured plasma volume and blood volume changes and may therefore represent an imprecise surrogate of intravascular congestion [2]. Bioelectrical impedance analysis (BIA) is a noninvasive method for assessing human body composition, including fluid distribution, and has been validated against deuterium oxide ingestion measurements [3]. BIA has shown promise in the diagnosis and management of heart failure [4] and we recently reported that the extracellular water–to–total body water (ECW/TBW) ratio measured by BIA was significantly correlated with Hb and B-type natriuretic peptide (BNP) levels in patients hospitalized in a cardiovascular ward [5]. Nevertheless, data directly examining the relationship between ePVS and ECW/TBW are scarce. Accordingly, we conducted a post hoc analysis of our recent dataset to investigate the association between these parameters.

## 2 Methods

We conducted a study of consecutive patients admitted to our cardiology department between January 1, 2021, and December 31, 2022. Among these, 851 patients met the inclusion criteria [5]. In the overall cohort, the most common primary diagnosis at admission was tachyarrhythmia (*n* = 361, 42.4%), followed by chronic coronary syndrome (CCS; *n* = 185, 21.7%) and valvular heart disease (*n* = 103, 12.1%). Consequently, only 4.9% (42/851) of patients were explicitly coded with ‘heart failure’ as their primary diagnosis. BIA was performed once at the time of hospitalization using the InBody770 (InBody Japan Inc., Tokyo, Japan) for all patients whenever feasible. ePVS was calculated using the Kaplan-Hakim formula based on laboratory data closest to the BIA date (Supplementary methods), as previously described [6]. PV expansion was defined as an ePVS >0%, and patient characteristics were compared according to the presence or absence of PV expansion. The association between ePVS and the extracellular water to ECW/TBW ratio was assessed using Spearman's rank correlation coefficient (ρ). The discriminative abilities of ECW/TBW and ePVS for identifying elevated B-type natriuretic peptide (BNP)—defined as a BNP value >200 pg/mL according to the guidelines of the Japanese Heart Failure Society—were evaluated using receiver operating characteristic (ROC) analysis and the DeLong test. These correlation and ROC analyses were also performed separately for each sex. Furthermore, within the subgroup of patients without PV expansion, clinical characteristics were compared between those with and without elevated ECW/TBW ratios based on the optimal cutoff value determined by the Youden index. Cases with any missing data were excluded from the final analysis. All statistical analyses were conducted using R software (version 4.4.1; R Foundation for Statistical Computing, Vienna, Austria).

## 3 Results

**Fig. 1:**
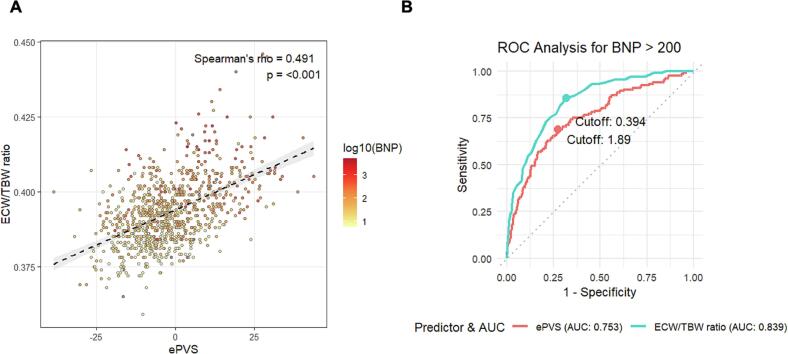
Relationship between estimated plasma volume status (ePVS), extracellular water to total body water ratio (ECW/TBW), and BNP levels. (A) A color-mapped scatter plot illustrating the association between ePVS and ECW/TBW. Color intensity represents plasma B-type natriuretic peptide (BNP) levels. A significant moderate positive correlation was identified between ePVS and ECW/TBW (Spearman's ρ = 0.491, *P* < 0.01). (B) Receiver operating characteristic (ROC) curves comparing the discriminative ability of ECW/TBW and ePVS for identifying elevated B-type natriuretic peptide (BNP > 200 pg/mL). ECW/TBW demonstrated superior performance, with an area under the curve (AUC) of 0.84 (95% CI: 0.81–0.87) compared with 0.75 (95% CI: 0.71–0.80) for ePVS.

Among 851, 344 patients (40.4%) exhibited PV expansion. Subjects with PV expansion showed significantly older age (65 vs 76, *p* < 0.01), higher ECW/TBW (0.389 vs 0.399, p < 0.01), higher cardio-thoracic ratio (49.7% vs 52.4%, p < 0.01), and higher BNP level (33.1 pg/mL vs 83.1 pg/mL) (Table 1). As shown in Fig. 1A, a significant moderate positive correlation was observed between ePVS and ECW/TBW (ρ = 0.491, *p* < 0.01). In ROC analysis, ECW/TBW demonstrated statistically superior performance for identifying elevated BNP, with an AUC of 0.84 (95% CI: 0.81–0.87) compared with 0.75 (95% CI: 0.71–0.80) for ePVS (*p* < 0.01) (Fig. 1B). Within the subgroup of patients without PV expansion (ePVS <0), those with an elevated ECW/TBW ratio (> 0.394) were significantly older, had a lower proportion of males, and exhibited higher BMI and body fat percentage (all *p* < 0.01; for details, see Table 2). Results stratified by sex were shown in Supplementary Fig. S1.

**Table 1:** Patient characteristics according to the presence of plasma volume expansion.

	PV expansion (−) *N* = 507	PV expansion (+) *N* = 344	*P*-value
Age, years	65 [56, 73]	76 [70, 81]	<0.001
Male (%)	171 (33.7)	157 (45.6)	<0.001
BMI, kg/m^2^	25.1 [22.6, 27.5]	21.4 [19, 23.3]	<0.001
TBW, L	35.3 [28.9, 40.3]	29.2 [24.1, 33.2]	<0.001
ECW, L	13.7 [11.28, 15.65]	11.6 [9.6, 13.2]	<0.001
ECW/TBW ratio	0.389 [0.383, 0.395]	0.399 [0.390, 0.406]	<0.001
Protein mass, kg	9.3 [7.6, 10.6]	7.5 [6.3, 8.7]	<0.001
Body fat percentage, %	0.30 [0.25, 0.36]	0.24 [0.2, 0.3]	<0.001
CTR, %	49.7 [46.6, 53.2]	52.4 [48, 57.4]	<0.001
LVEF, %	62.3 [56.1, 67.3]	61.0 [52.0, 67.2]	0.063
Hb, g/dl	14.3 [13.5, 15.4]	11.9 [10.8, 13]	<0.001
Hct, %	42.8 [40.7, 45.9]	36.1 [33, 39]	<0.001
aPV, mL	2381 [2157, 2623]	2256 [1959, 2503]	<0.001
iPV, mL	2620[2356, 2941]	2062 [1779, 2285]	<0.001
ePVS, %	−8.55 [−13.75, −4.62]	7.50 [3.61, 14.0]	<0.001
eGFR, mL/min/1.73m^2^	64.9 [54.4, 77.7]	55.9 [40.1, 71.7]	<0.001
BNP, pg/mL	33.1 [12.7, 78.4]	83.1 [34.3, 227.3]	<0.001

TBW: Total Body Water, ECW: Extracellular Water, BMI: Body Mass Index, CTR: Cardiothoracic Ratio, LVEF: Left Ventricular Ejection Fraction, Hb: Hemoglobin, Hct: Hematocrit, aPV: actual Plasma Volume, iPV: ideal Plasma Volume, ePVS: estimated Plasma Volume Status, eGFR: estimated Glomerular Filtration Rate, BNP: B-type Natriuretic Peptide.

**Table 2:** Patient characteristics according to the presence of elevated extracellular volume among patients without plasma volume expansion.

	ECW/TBW ≤ 0.394 *N* = 379	ECW/TBW > 0.394 *N* = 128	P-value
Age, years	63.00 [54.75, 71.00]	74.00 [66.00, 79.25]	<0.01
Male (%)	274 (72.9)	60 (46.9)	<0.01
BMI, kg/m^2^	24.75 [22.29, 26.99]	26.02 [23.74, 28.10]	<0.01
TBW, L	36.00 [30.48, 40.80]	31.65 [27.08, 38.25]	<0.01
ECW, L	13.85 [11.82, 15.79]	12.55 [10.88, 15.24]	0.01
ECW/TBW ratio	0.39 [0.38, 0.39]	0.40 [0.40, 0.40]	<0.01
Protein mass, kg	9.50 [8.00, 10.80]	8.20 [7.00, 10.00]	<0.01
Body fat percentage, %	0.29 [0.24, 0.34]	0.36 [0.29, 0.40]	<0.01
CTR, %	48.90 [46.05, 52.35]	52.20 [49.20, 55.62]	<0.01
LVEF, %	61.70 [56.00, 66.90]	64.10 [56.55, 68.00]	0.09
Hb, g/dl	14.60 [13.70, 15.60]	13.60 [12.90, 14.43]	<0.01
Hct, %	43.30 [41.30, 46.50]	41.00 [39.27, 43.62]	<0.01
aPV, mL	2377.02 [2155.27, 2584.02]	2434.11 [2199.62, 2688.98]	0.20
iPV, mL	2632.50 [2359.00, 2940.60]	2606.55 [2344.77, 2935.72]	0.55
ePVS, %	−9.20 [−14.96, −4.97]	−7.03 [−11.05, −3.78]	<0.01
eGFR, mL/min/1.73m^2^	67.60 [56.30, 79.08]	60.50 [48.88, 69.88]	<0.01
BNP, pg/mL	25.75 [10.30, 64.25]	68.70 [24.90, 155.68]	<0.01

TBW: Total Body Water, ECW: Extracellular Water, BMI: Body Mass Index, CTR: Cardiothoracic Ratio, LVEF: Left Ventricular Ejection Fraction, Hb: Hemoglobin, Hct: Hematocrit, aPV: actual Plasma Volume, iPV: ideal Plasma Volume, ePVS: estimated Plasma Volume Status, eGFR: estimated Glomerular Filtration Rate, BNP: B-type Natriuretic Peptide.

## 4 Discussion

Accurate assessment of volume status is essential for the diagnosis and treatment of heart failure, as inappropriate volume management is closely linked to clinical outcomes [7]. In general, approximately one-third of TBW is distributed within the extracellular compartment. ECW consists of interstitial fluid and plasma, with roughly 75% of extracellular volume being interstitial and the remaining 25% plasma [8]. In congestive heart failure, expansion of both interstitial fluid and plasma leads to an increased proportion of ECW (Supplementary Fig. S2); however, some patients exhibit increased interstitial fluid without PV expansion; therefore, BIA, which reflects extracellular fluid distribution, may provide superior diagnostic value for detecting elevated BNP levels. Our finding that elevated ECW/TBW without PV expansion was predominantly observed in elderly women with higher body fat is consistent with previous work by Waki et al., who reported that obese women exhibited a higher ECW ratio compared with their non-obese counterparts [9]. As relative blood volume status significantly influences the response to diuretic therapy, treatment responsiveness may differ depending on the presence of plasma volume expansion (or body fat percentage), even among patients with the same BNP or ECW/TBW levels. Therefore, combining ePVS with BIA may provide a more comprehensive assessment of congestion in patients with heart failure.

## 5 Limitations

This study is limited by its retrospective design and the use of primary admission diagnoses for disease categorization. This approach likely results in an underestimation of the actual prevalence of heart failure within our cohort, as only 4.9% (42/851) of patients were explicitly coded with ‘heart failure’ as their primary diagnosis.

## CRediT authorship contribution statement

**Ryo Miyabe:** Writing – review & editing, Investigation, Formal analysis, Conceptualization. **Akinori Higaki:** Writing – original draft, Methodology, Conceptualization. **Keisho Kurokawa:** Writing – review & editing. **Tomoaki Nishikawa:** Writing – review & editing, Formal analysis, Data curation. **Rikako Horie:** Writing – review & editing. **Yasuhisa Nakao:** Writing – review & editing. **Tomoki Fujisawa:** Writing – review & editing. **Yusuke Akazawa:** Writing – review & editing. **Toru Miyoshi:** Writing – review & editing. **Hiroshi Kawakami:** Writing – review & editing. **Haruhiko Higashi:** Writing – review & editing. **Shunsuke Tamaki:** Writing – review & editing. **Kazuhisa Nishimura:** Writing – review & editing. **Katsuji Inoue:** Writing – review & editing. **Shuntaro Ikeda:** Writing – review & editing. **Osamu Yamaguchi:** Writing – review & editing, Supervision.

## Ethical statement

The present study was approved by the Research Ethics Committee of Ehime University Graduate School of Medicine (IRB2310003), which waived the requirement for consent. This is a retrospective study using de-identified data, and a waiver of consent was granted by the research ethics boards.

Ryo Miyabe, Akinori Higaki, Keisho Kurokawa, Tomoaki Nishikawa, Rikako Horie, Yasuhisa Nakao, Tomoki Fujisawa, Yusuke Akazawa, Toru Miyoshi, Hiroshi, Kawakami, Haruhiko Higashi, Shunsuke Tamaki, Kazuhisa Nishimura, Katsuji Inoue, Shuntaro Ikeda, Osamu Yamaguchi.

Department of Cardiology, Pulmonology, Hypertension & Nephrology, Ehime.

University Graduate School of Medicine, Toon, Japan.

## Declaration of competing interest

The authors declare that they have no known competing financial interests or personal relationships that could have appeared to influence the work reported in this paper.

## Appendix A Supplementary data

Supplementary Methods: Calculation of estimated plasma volume status (ePVS).
