# Supplementary material for: Correlation between estimated plasma volume status and extracellular volume ratio determined by bioelectrical impedance analysis in cardiovascular disease patients
Source: Am Heart J Plus. 2026 May 11;66:100794. doi: 10.1016/j.ahjo.2026.100794 (PMC13195339; doi:10.1016/j.ahjo.2026.100794)
Supplement: Supplementary file 1 — Supplementary Methods: Calculation of estimated plasma volume status (ePVS). [file mmc1.docx]

**Supplementary Methods: Calculation of estimated plasma volume status (ePVS)**

The estimated plasma volume status (ePVS) was calculated using the Kaplan-Hakim formula based on laboratory data. Specifically, actual PV (mL) was defined as (1−Hct)*[a+(b*BW)], where a=1,530 and b=41.0 for males, and a=864 and b=47.2 for females.  Ideal PV (mL) was defined as c×BW, where c=39 for males and c=40 for females. Subsequently, ePVS was determined as [(actual PV−ideal PV)/ideal PV]*100, representing the percentage deviation of actual PV from the ideal PV.
