# Supplementary material for: Correlation between estimated plasma volume status and extracellular volume ratio determined by bioelectrical impedance analysis in cardiovascular disease patients
Source: Am Heart J Plus. 2026 May 11;66:100794. doi: 10.1016/j.ahjo.2026.100794 (PMC13195339; doi:10.1016/j.ahjo.2026.100794)
Supplement: Supplementary file 2 — Supplementary figures [file mmc2.pdf]

Supplementary Figure S1.

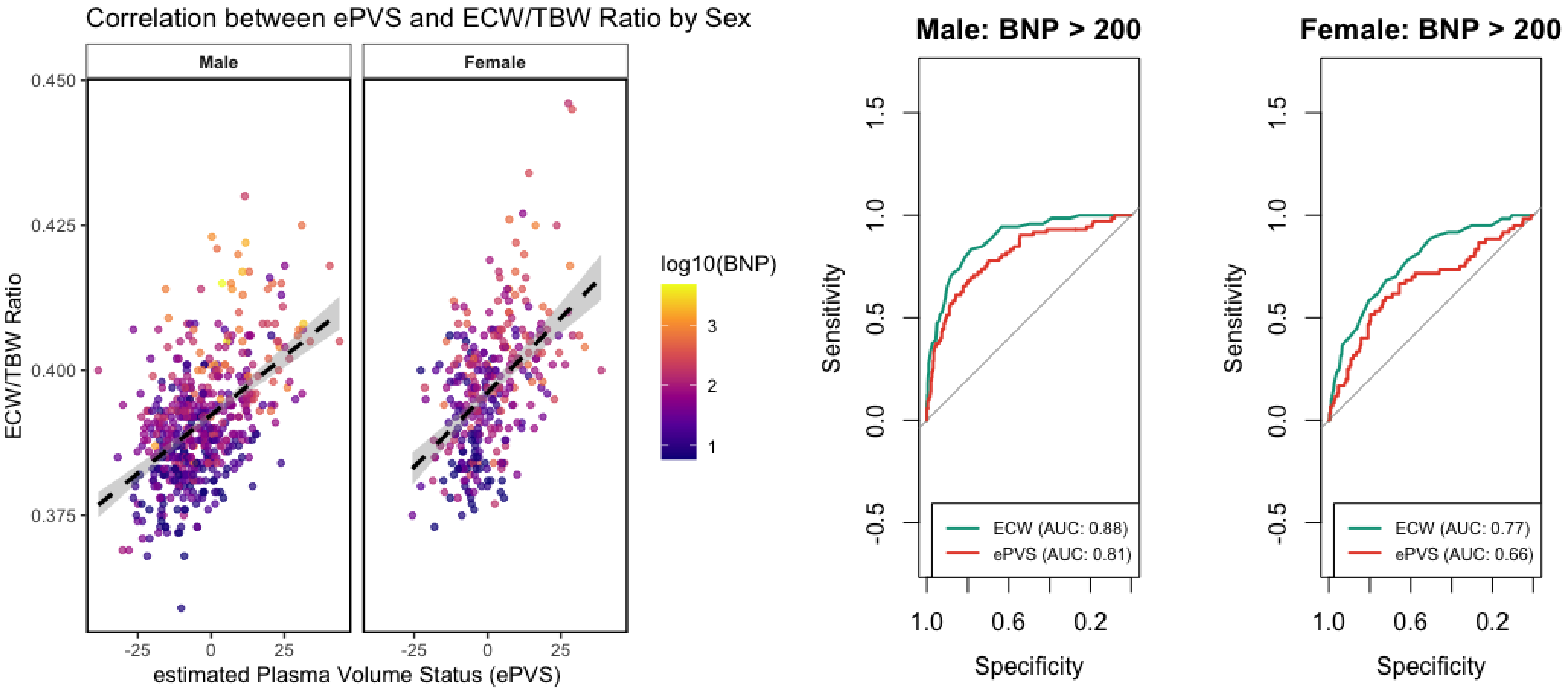

Supplementary Figure S2.

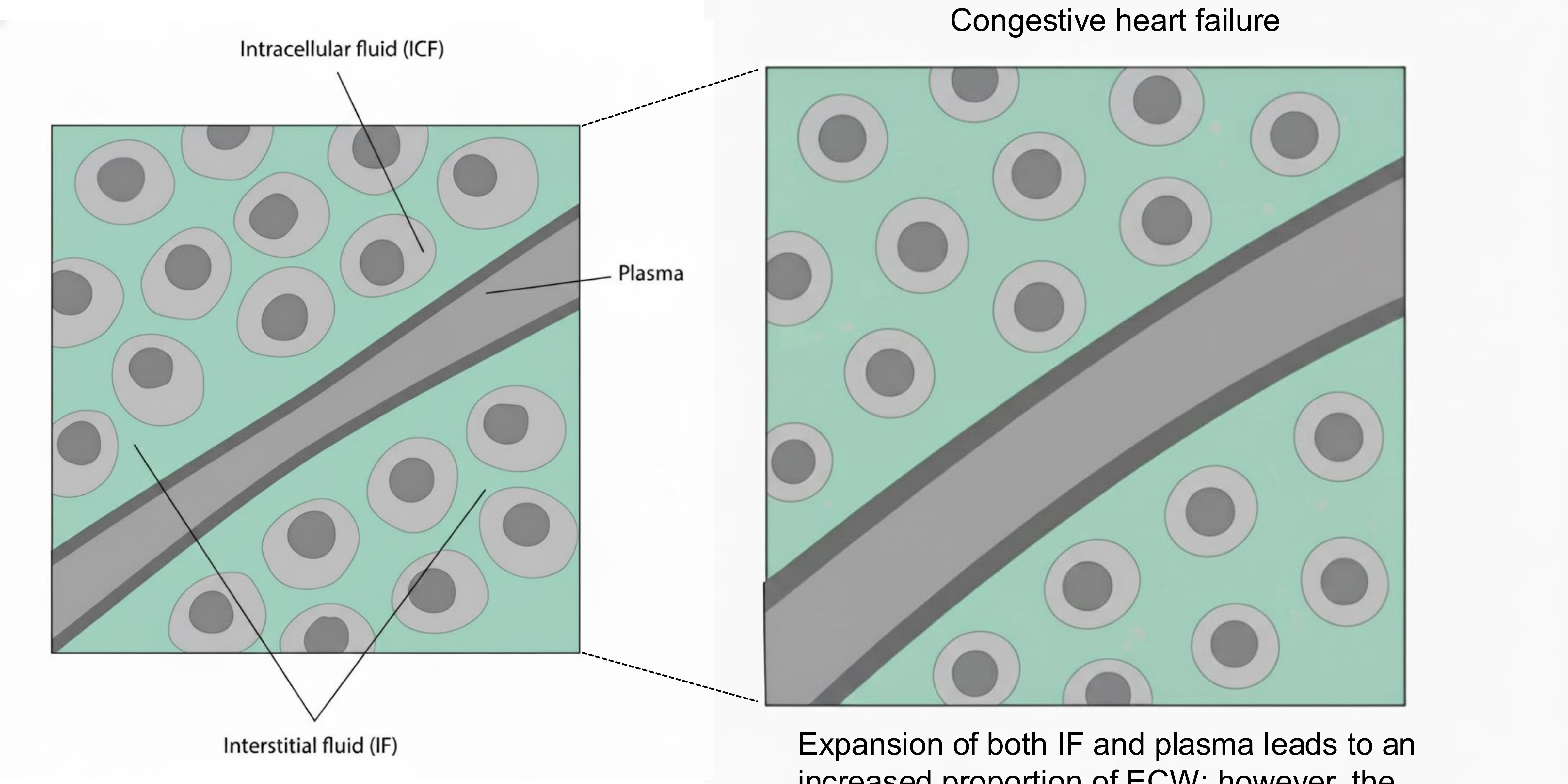

Extracellular water = Plasma volume + IF

Expansion of both IF and plasma leads to an increased proportion of ECW; however, the increase in IF is typically more pronounced.
